# 

**Published:** 1877-08

**Authors:** 


### Table of Contents.

### ORIGINAL COMMUNICATIONS.

Nasal Catarrh. *By J. C. LeHardy, M.D., Savannah, Ga.* .......... 257

What is Cincho-Quinine? *By Walter A. Taylor, Ph. G., Atlanta, Ga.* 264

The New Chemical Notation. *By J. H. Logan, A.M., M.D., Atlanta, Ga.* .......... 269

Meningitis. *By C. A. Hentz, M.D., Quincy, Fla.* .......... 275

### SELECTIONS.

Trismus Nascentium .......... 277
Danger from Hypodermic Injections .......... 279
Acute Rheumatism Treated with Salicylic Acid .......... 282
A Case of Successful Extirpation of the Parotid Gland .......... 285
Experience in Puerperal Eclampsia .......... 287
Remarks on the Best Methods of Promoting Post-Partum Uterine Contractions .......... 294
A Case of Traumatic Tetanus in a Young Child, with Treatment and Recovery .......... 297

### REPORTS OF SOCIETIES.

Pathological Society of Philadelphia .......... 302
St. Louis Medical Society .......... 306

### EDITORIAL.

To our Contributors .......... 314
Editorial Correspondence .......... 316
Erratum .......... 319

### EXCERPTA.

To Relieve Morbid Thirst for Alcoholic Drinks .......... 319
Simple Mode of Checking Epistaxis .......... 320
Remedies for Sleeplessness .......... 320
To Physicians .......... 320

